# Exploring Multidimensional Diabetes Self-Management and Health-Related Functioning in Relation to Vascular Complications

**DOI:** 10.3390/jcm15156125

**Published:** 2026-08-06

**Authors:** Claudiu Cobuz, Mădălina Ungureanu-Iuga, Iuliana Costescu, Alina Cornea, Maricela Cobuz

**Affiliations:** 1 Faculty of Medicine and Biological Sciences, Ştefan cel Mare University of Suceava, 13th Universităţii Street, 720229 Suceava, Romania; claudiu.cobuz@usm.ro (C.C.); iuliana.costescu1@student.usv.ro (I.C.); alina.cornea1@student.usv.ro (A.C.); cobuz.maricela@spjsv.ro (M.C.); 2 Integrated Center for Research, Development and Innovation in Advanced Materials, Nanotechnologies, and Distributed Systems for Fabrication and Control (MANSiD), Ştefan cel Mare University of Suceava, 13th Universităţii Street, 720229 Suceava, Romania; 3 “Sfântul Ioan cel Nou” Emergency Clinical Hospital, 720224 Suceava, Romania

**Keywords:** type 2 diabetes mellitus, life quality, diabetes complications, chronic disease distress, functional limitations

## Abstract

**Background/Objectives**: Diabetes mellitus is associated with complex behavioral, psychosocial, and functional challenges that may influence disease burden and complication profiles. This study aimed to identify latent dimensions of diabetes self-management and health-related functioning and to evaluate their association with the type of diabetes-related complications. **Methods**: A cross-sectional study was conducted in 300 patients with type 2 diabetes, classified into microvascular and macrovascular complication groups. A questionnaire comprising DSMQ (diabetes self-management questionnaire) derived items, SF-36-derived items, and other questions was administered to all participants. Exploratory factor analysis was performed to identify latent dimensions related to self-management, emotional burden, dietary behavior, glycemic control challenges, glucose monitoring, and functional status. Composite scores were calculated for factors with acceptable internal consistency. Between-group comparisons, Spearman correlation analysis, and binary logistic regression models adjusted for age, sex, residence area, marital status, education, diabetes duration, and treatment type were applied. **Results**: Six latent factors were identified: Emotional Burden, Dietary Self-Management, Functional Limitations, Glycemic Control Challenges, Glucose Monitoring, and General Health Perception. However, only the factors demonstrating acceptable internal consistency were retained for subsequent analyses: Emotional Burden, Functional Limitations, and Glycemic Control Challenges. Functional Limitations showed the strongest association with complication type, with patients with macrovascular complications reporting greater functional impairment. After adjustment for potential confounders and correction for multiple comparisons, higher adherence to the recommended meal plan (DSMQ2) and greater functional limitations remained the principal differences between the complication groups. Functional Limitations Score remained independently associated with complication type in both unadjusted and adjusted logistic regression models. **Conclusions**: These cross-sectional findings support the integration of functional, psychosocial, and behavioral assessment into comprehensive diabetes care and highlight differences in patient-reported outcomes across complication groups.

## 1. Introduction

Diabetes mellitus is a chronic metabolic disease associated not only with hyperglycemia, but also with a wide range of behavioral, psychosocial, and functional challenges. Effective diabetes care requires continuous self-management, including adherence to pharmacological treatment, glucose monitoring, dietary regulation, physical activity, recognition of hypo- and hyperglycemia, and adaptation of daily behaviors to long-term disease demands [1,2].

Diabetes-related complications are traditionally classified into microvascular complications, such as retinopathy, nephropathy, and neuropathy, and macrovascular or cardiovascular complications, including coronary artery disease, cerebrovascular disease, heart failure, and peripheral arterial disease [3]. Although these complications share common metabolic and vascular risk factors, they may be associated with different patient profiles, levels of functional impairment, emotional burden, and self-management difficulties [4]. Diabetes-related complications have been consistently associated with impaired health-related quality of life, but previous studies suggest that the magnitude and profile of this impairment differ according to complication type [5]. In the DISCOVER prospective cohort, macrovascular complications and neuropathy were among the complications most strongly associated with deterioration in quality of life, supporting the relevance of evaluating functional and patient-reported outcomes beyond glycemic parameters [5].

Self-management in diabetes is a multidimensional construct. Beyond glycemic control, patient outcomes are influenced by psychological distress, perceived disease burden, dietary behaviors, confidence in self-care, and the ability to maintain daily functioning. Therefore, a purely biomedical approach may fail to capture important patient-centered dimensions that contribute to disease burden and clinical outcomes [1,6]. Longitudinal evidence also indicates that incident diabetes complications, including stroke, heart failure, myocardial infarction, renal failure, blindness, and amputation, are associated with measurable declines in EQ-5D utility scores [7]. This supports the assumption that diabetes complications should not be interpreted only as clinical endpoints, but also as determinants of functional status and daily-life impairment. Previous research has linked diabetes self-management behaviors with microvascular complications, suggesting that inadequate self-care could be associated with vascular complications of diabetes [8]. However, because these relationships are likely bidirectional, particularly in established disease, the temporal sequence between self-management behaviors and complications remains uncertain. Most available studies have examined self-management domains separately rather than integrating behavioral, emotional, and functional dimensions into a unified analytical framework. Psychological distress is another clinically relevant dimension, as diabetes-related distress has been associated with poorer self-care, higher HbA1c, and increased concern regarding complications [9]. Studies examining cardiovascular risk have also shown that diabetes-related distress may be linked to cardiovascular complications, highlighting the need to explore psychosocial burden in relation to complication profiles [10].

The Diabetes Self-Management Questionnaire and its revised versions were developed to assess multiple self-care behaviors, including glucose monitoring, dietary control, medication adherence, physical activity, and interaction with healthcare professionals [11]. In the present study, selected concepts and items derived from the DSMQ were incorporated into a broader questionnaire together with health-functioning items derived from the SF-36 and additional questions developed. These instruments support the concept that diabetes self-management is multidimensional, but further studies are needed to determine how latent self-management profiles relate to specific complication patterns. Exploratory factor analysis allows the identification of latent domains underlying complex questionnaire-based data. When combined with regression modeling, this approach may help identify clinically relevant profiles associated with different patterns of diabetes-related complications.

The novelty of the present study lies in the integrated multidimensional evaluation of diabetes self-management and health-related functioning in patients with diabetes-related complications. Specifically, this study aimed to identify latent psychosocial, behavioral, and functional dimensions and to assess their association with microvascular and macrovascular complication profiles.

## 2. Materials and Methods

### 2.1. Study Design, Participants, and Questionnaire Development

**Figure 1:**
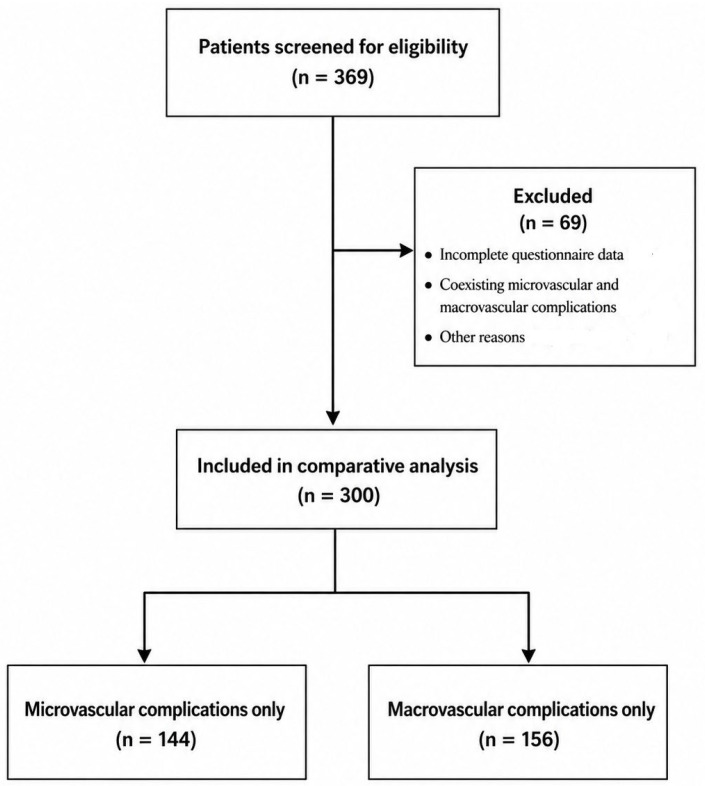
Flow diagram for participant screening.

A cross-sectional observational study was conducted in May 2026 and included 300 adults diagnosed with type 2 diabetes. A total of 369 patients were screened (Figure 1). Of these, 69 were excluded: 26 for incomplete questionnaire data, 32 for coexisting microvascular and macrovascular complications, and 11 for other eligibility-related reasons. The remaining 300 participants were included in the comparative analysis, comprising 144 patients with microvascular complications and 156 patients with macrovascular complications. The population was 35,217 patients with type 2 diabetes, and the sample size of 300 participants corresponds to an estimated margin of error of 5.6% at the 95% confidence level.

Participants were evaluated within the Department of Diabetes, Nutrition and Metabolic Diseases, as well as in the related specialty outpatient clinic, within the “Sfântul Ioan cel Nou” County Emergency Clinical Hospital in Suceava. Participants completed a questionnaire designed to assess diabetes self-management behaviors, dietary practices, perceived health, functional limitations, psychosocial burden, and the perceived impact of diabetes on daily life. The questionnaire included selected items and concepts derived from previously published instruments [11,12], together with additional questions developed for the objectives of the present study. Participants were recruited using a consecutive, non-probability sampling approach. All potentially eligible adults with type 2 diabetes attending the Department of Diabetes, Nutrition and Metabolic Diseases and its affiliated outpatient clinic during the recruitment period were screened in the order in which they presented.

The inclusion criteria in the study were: age ≥ 18 years, confirmed diagnosis of type 2 diabetes, and voluntary agreement to participate in the research. Patients diagnosed with type 1 diabetes or gestational diabetes were excluded, as well as people who provided incomplete answers to the questionnaire. Data were collected directly from participants by individually addressing the questions included in the questionnaire (Supplementary Materials). Before applying it, the participants were presented with the purpose of the study, the voluntary nature of participation, and the confidentiality of the data provided. The information collected included socio-demographic and clinical data, such as age, sex, residential area, educational level, duration of diabetes, type of treatment followed, and the presence of complications associated with diabetes. Also, eating behaviors, the level of diabetes self-management, perception of one’s own health status, the level of stress associated with managing the disease, and the impact of diabetes on quality of life were assessed. To assess diabetes self-management and quality of life, items adapted from the validated instruments DSMQ (Diabetes Self-Management Questionnaire) [11] and SF-36 (Short Form Health Survey) [12] were used. The study questionnaire, including item wording, response options, and coding rules, is provided in the Supplementary Questionnaire and Supplementary Table S4.

### 2.2. Definition of Complication Groups

Differences between patients with microvascular and macrovascular diabetes complications were analyzed. Microvascular complications were defined as the presence of diabetic retinopathy, diabetic nephropathy, and/or diabetic neuropathy, based on available clinical records. Macrovascular complications were defined as a documented history of coronary artery disease, myocardial infarction, stroke, heart failure, peripheral arterial disease, or other clinically confirmed macrovascular disease. Among the 300 participants included in the study, cardiovascular disease was the most frequently reported complication (51.7%), followed by nephropathy (20.3%), neuropathy (18.0%), retinopathy (8.0%), and other microvascular complications (2.0%). Classification was based on the documented complication profile recorded in the clinical records. Participants with documented complications belonging to both categories were not classified according to a predominant complication profile but were excluded from the comparative analysis.

### 2.3. Statistical Analysis of Data

Statistical analysis was performed using IBM SPSS Statistics software, trial version (IBM Corp., Armonk, NY, USA). Descriptive statistics were used to summarize demographic and clinical characteristics of the study population. Continuous demographic and clinical variables with skewed distributions were summarized as medians and interquartile ranges. Differences between the two complication groups were initially assessed using separate unadjusted general linear models (ANOVA). To compare questionnaire outcomes between the two complication groups while accounting for potential confounding variables, analyses of covariance (ANCOVA) were performed, with complication group entered as the fixed factor and age, sex, residence area, marital status, educational level, diabetes duration, and diabetes treatment included as covariates. ANCOVA was considered an appropriate approach for comparing adjusted group means across multiple patient-reported outcomes. Unadjusted means, adjusted estimated marginal means, F statistics, and corresponding *p* values were reported for each outcome variable. A two-sided *p* value < 0.05 was considered statistically significant. Statistical significance was established at *p* ≤ 0.05. To account for multiple comparisons, *p*-values from the adjusted ANCOVA models were corrected using the Benjamini–Hochberg false discovery rate (FDR) procedure. As a sensitivity analysis, the questionnaire outcomes that remained statistically significant after Benjamini–Hochberg correction were additionally evaluated using ordinal logistic regression models adjusted for the same covariates.

Before exploratory factor analysis, all reverse-scored items (DSMQ3, DSMQ6, confidence in self-management, support and information need, and difficulty following the diet) were recoded to ensure a consistent scoring direction within each construct before factor extraction. The recoded variables were subsequently used to calculate composite scores. The suitability of the dataset for exploratory factor analysis (EFA) was assessed using the Kaiser–Meyer–Olkin (KMO) measure of sampling adequacy and Bartlett’s test of sphericity. EFA was performed using Principal Axis Factoring with Promax rotation and Kaiser normalization to identify latent dimensions related to diabetes self-management, psychosocial burden, dietary behaviors, and functional status. The number of retained factors was determined based on eigenvalues (>1), inspection of the scree plot, and the interpretability of the factor solution. Factor loadings ≥ 0.40 were considered meaningful for factor interpretation. Because only selected DSMQ items relevant to the objectives of the present study were administered rather than the complete validated instrument, the original DSMQ scoring algorithm was not applied. Exploratory factor analysis was therefore performed to examine the latent structure of the selected items within the study sample, and composite scores were subsequently calculated by summing the items loading on each factor. Exploratory factor analysis was performed on the correlation matrix, which standardizes variables irrespective of their original numerical ranges. Composite factor scores were subsequently calculated by aggregating variables with the highest loadings within each extracted factor. Before constructing the composite scores, items formulated in the opposite direction of the underlying construct were reverse-coded to ensure a consistent scoring direction. Composite scores were then calculated by aggregating the items retained for each exploratory dimension identified by the EFA. Consequently, higher values consistently represent higher levels of the construct reflected by each composite score (e.g., greater emotional burden, greater functional limitations, or greater glycemic control challenges). Composite scores were calculated as the arithmetic mean of the retained items within each factor after reverse coding where applicable. Specifically, the Emotional Burden Score was calculated as the mean of fatigue, sadness, social life impairment, diabetes impact on life, diabetes-related stress level, and stress-related eating. The Functional Limitations Score was calculated as the mean of severe limitations, moderate limitations, mobility limitations, and work impairment. The Glycemic Control Challenges Score was calculated as the mean of DSMQ5, reverse-coded DSMQ6, DSMQ7, hyperglycemia, and reverse-coded confidence in self-management. Because the confidence in self-management item used a broader response scale (1–10) than the remaining items (1–4 and 1–2), it was first reverse-coded and then linearly standardized to a 1–4 scale to ensure comparability with the other retained items before calculating the composite score as the arithmetic mean of the five items. Associations between factor scores were evaluated using Spearman’s rank correlation coefficient. Binary logistic regression analyses were further conducted to investigate the association between the derived factor scores and complication type (microvascular vs. macrovascular complications). Both unadjusted and adjusted models were calculated. The dependent variable was complication type (microvascular complications = 1; macrovascular complications = 0). Each composite score was evaluated in a separate logistic regression model. Adjusted models included age, sex, residence area, marital status, education level, diabetes duration, and treatment type as covariates. Odds ratios (ORs) and 95% confidence intervals (95% CIs) were reported for all regression analyses.

## 3. Results

### 3.1. Characteristics of Groups

No significant differences were observed between the microvascular and macrovascular complications groups regarding sex (*p* = 0.148), residence area (*p* = 0.477), marital status (*p* = 0.816), education level (*p* = 0.538), age (*p* = 0.095), or diabetes duration (*p* = 0.156). However, a statistically significant difference was identified for treatment type (*p* = 0.012), with patients in the macrovascular complications group more frequently receiving oral antidiabetic medication, whereas mixed treatment and insulin therapy were more common among participants with microvascular complications (Table 1).

In the unadjusted analysis, several variables differed significantly between patients with microvascular and macrovascular complications (Table 2). DSMQ2—adherence to the recommended meal plan was significantly higher in the microvascular group than in the macrovascular group (3.077 vs. 2.724; *F* = 8.382, *p* = 0.004). Significant differences were also observed for Blood glucose measurement (*F* = 4.820, *p* = 0.029), with higher scores in the microvascular group (1.986 vs. 1.788), and Hypoglycemia (*F* = 5.530, *p* = 0.019), where participants with macrovascular complications reported higher hypoglycemia scores (1.827 vs. 1.704). Regarding dietary behaviors, the fruit consumption score was significantly higher in the microvascular group (1.570 vs. 1.359; *F* = 8.430, *p* = 0.004). Functional status also differed between groups, with patients with macrovascular complications reporting significantly greater Severe limitations (*F* = 9.764, *p* = 0.002), Moderate limitations (*F* = 8.432, *p* = 0.004), and Mobility limitations (*F* = 18.451, *p* ≤ 0.001). In addition, the microvascular group reported higher Energy level scores (*F* = 6.560, *p* = 0.011), higher Perceived disease control (*F* = 4.018, *p* = 0.046), and greater Diabetes impact on life (*F* = 5.155, *p* = 0.024). No significant differences were found for the remaining variables.

After adjustment for covariates, most of the significant findings remained stable. DSMQ2—adherence to the recommended meal plan continued to be significantly higher in the microvascular group (adjusted means: 3.096 vs. 2.714; *F* = 9.097, *p* = 0.003). Hypoglycemia also remained significantly higher in the macrovascular group (*F* = 4.895, *p* = 0.028), while Fruit consumption remained significantly higher in the microvascular group (*F* = 6.785, *p* = 0.010). Additionally, Stress-related eating, which had shown only a trend toward significance in the unadjusted analysis (*p* = 0.059), became statistically significant after adjustment (*F* = 4.064, *p* = 0.045), with higher adjusted scores in the microvascular group (2.887 vs. 2.532). The differences in functional status also persisted after adjustment, with patients with macrovascular complications continuing to report higher Severe limitations (*F* = 10.336, *p* = 0.001), Moderate limitations (*F* = 7.850, *p* = 0.005), and Mobility limitations (*F* = 14.817, *p* ≤ 0.001). Likewise, Energy level remained significantly higher in the microvascular group (*F* = 7.227, *p* = 0.008), as did Diabetes impact on life (*F* = 5.280, *p* = 0.022). Adjustment for covariates modified the significance of two variables. Blood glucose measurement and perceived disease control, which were significant in the unadjusted analysis, were no longer statistically significant after adjustment (*p* = 0.119 and *p* = 0.156, respectively). Conversely, Stress-related eating became significant only after adjustment, suggesting that controlling for potential confounders revealed an independent association with the type of diabetes complication. After adjustment for age, sex, residence area, marital status, education, diabetes duration, and treatment type, *p*-values were further corrected for multiple testing using the Benjamini–Hochberg false discovery rate procedure. Following correction, significant differences remained for DSMQ2 (adherence to the recommended meal plan), Moderate limitations, Severe limitations, and Mobility limitations. Sensitivity analyses using adjusted ordinal logistic regression yielded results consistent with the primary ANCOVA analyses. Adherence to the recommended meal plan (DSMQ2) (Wald = 7.90, *p* = 0.005), severe limitations (Wald = 9.65, *p* = 0.002), moderate limitations (Wald = 7.08, *p* = 0.008), and mobility limitations (Wald = 15.53, *p* < 0.001) remained significantly associated with complication group. Although hypoglycemia, fruit consumption, moderate limitations, energy level, diabetes impact on life, and stress-related eating showed nominal statistical significance before correction, these associations did not remain significant after controlling for multiple comparisons and should therefore be interpreted as exploratory findings.

### 3.2. Exploratory Factor Analysis and Composite Scores

The KMO value was 0.745, indicating moderate sampling adequacy (Table S2 Supplementary Materials). Bartlett’s test of sphericity was highly significant (χ^2^ = 4323.697, df = 903, *p* ≤ 0.001), supporting the suitability of the correlation matrix for exploratory factor analysis. The retained factors explained 42.80% of the total variance (complete eigenvalues and explained variance are provided in the Supplementary Materials). Principal Axis Factoring with Promax rotation identified a six-factor structure reflecting distinct but interrelated dimensions of diabetes self-management and health-related functioning. Factor 1 represented Emotional Burden, with high loadings for fatigue, sadness, social life impairment, stress-related eating, diabetes impact on life, and diabetes-related stress level. Factor 2 reflected Dietary Self-Management, including carbohydrate awareness, diet adjustment based on glucose levels, DSMQ-related dietary items, sweets consumption, and perceived diet adequacy. Factor 3 represented Functional Limitations, with high loadings for moderate limitations, mobility limitations, severe limitations, and work impairment. Factor 4 was interpreted as Glycemic Control Challenges, including hyperglycemia, diabetes self-management items, and confidence-related indicators. Factor 5 was characterized by Glucose Monitoring behaviors, particularly blood glucose measurement and DSMQ item 1. Factor 6 reflected General Health Perception, with general health status emerging as the most prominent variable. Thus, exploratory factor analysis identified six exploratory dimensions: Emotional Burden, Dietary Self-Management, Functional Limitations, Glycemic Control Challenges, Glucose Monitoring, and General Health Perception.

Reliability analysis of the factors revealed acceptable internal consistency for Factor 1—Emotional Burden (Cronbach’s alpha = 0.7), good internal consistency for Factor 3—Functional Limitations (Cronbach’s alpha = 0.8), and limited internal consistency for Factor 4—Glycemic Control Challenges (Cronbach’s alpha = 0.6). The remaining factors were heterogeneous and were not used for composite score calculation. Factor scores were calculated by aggregating the items with the highest loadings within each extracted factor, generating composite scores for Emotional Burden, Functional Limitations, and Glycemic Control Challenges.

Spearman correlation analysis showed weak but statistically significant associations between the derived composite scores. Emotional Burden Score was negatively correlated with Functional Limitations Score (ρ = −0.210, *p* ≤ 0.01) and positively correlated with Glycemic Control Challenges Score (ρ = 0.205, *p* ≤ 0.01). Functional Limitations Score was also negatively correlated with Glycemic Control Challenges Score (ρ = −0.122, *p* ≤ 0.01), but the correlation was weak. These findings suggest that the latent dimensions identified by EFA are moderately interrelated (Table 3).

**Figure 2:**
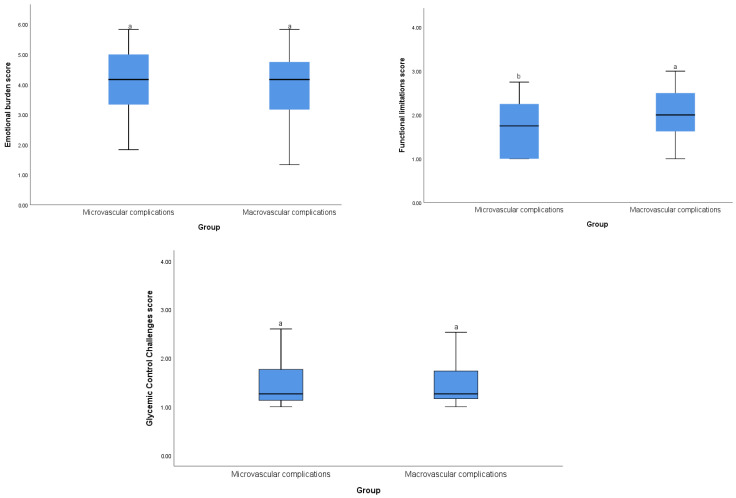
Distribution of the retained composite scores according to complication group. The center line represents the median, the box indicates the interquartile range (25th–75th percentiles), and whiskers extend to the most extreme observations within 1.5 × IQR. Different superscript letters (a, b) indicate statistically significant differences between groups after adjusted ANCOVA. Higher scores represent higher levels of the corresponding construct.

Figure 2 shows that the Functional Limitations Score was significantly higher in patients with macrovascular complications compared with those with microvascular complications, whereas the Emotional Burden and Glycemic Control Challenges scores did not differ significantly between groups. After adjustment for age, sex, residence area, marital status, education, diabetes duration, and treatment, only Functional Limitations remained significantly associated with complication group after Benjamini–Hochberg correction (*F* = 11.676, *p* = 0.001, FDR-adjusted *p* = 0.003, *partial η*^2^ = 0.039), representing a small effect size. No significant associations were observed for emotional burden (*F* = 3.207, *p* = 0.074, FDR-adjusted *p* = 0.111, *partial η*^2^ = 0.011) or glycemic control (*F* = 0.878, *p* = 0.350, FDR-adjusted *p* = 0.350, *partial η*^2^ = 0.003).

### 3.3. Logistic Regression Analysis of Diabetes Complication Type

Binary logistic regression showed that Functional Limitations Score was significantly associated with complication type. After adjustment for demographic and clinical covariates, a higher Functional Limitations Score was associated with lower odds of belonging to the microvascular complications group and, consequently, with a higher probability of being classified in the macrovascular complications group. Emotional Burden Score and Glycemic Control Challenges Score were not significantly associated with complication type in either unadjusted or adjusted analyses (Table 4).

All adjusted binary logistic regression models were statistically significant (Table S3) according to the likelihood ratio test (*p* ≤ 0.05). Pearson goodness-of-fit tests were non-significant, indicating no evidence of poor model fit, whereas Deviance tests were interpreted cautiously because of the large number of sparse subpopulations. *Pseudo-R*^2^ values (Nagelkerke: 0.106–0.148) suggested modest explanatory power, which is expected in observational clinical studies.

## 4. Discussion

The present study identified a multidimensional structure underlying diabetes self-management and health-related functioning in patients with diabetes-related complications. The results showed that after adjustment for confounding variables, patients with macrovascular complications exhibited significantly greater Severe limitations, Moderate limitations, and Mobility limitations than those with microvascular complications. This finding is consistent with previous studies reporting that macrovascular complications, particularly ischemic heart disease and stroke, are among the strongest determinants of impaired physical functioning and reduced health-related quality of life in people with diabetes [13]. Other studies demonstrated that hypoglycemia is associated with increased cardiovascular vulnerability, poorer quality of life, and a greater burden of disease in patients with diabetes [14]. Higher hypoglycemia scores were observed in the adjusted analyses; however, this association did not remain statistically significant after correction for multiple comparisons. Previous research indicated that autonomic dysfunction is associated with a higher burden of diabetic complications and may contribute to poorer cardiovascular and renal outcomes [15,16]. Although patients with microvascular complications showed higher Energy level scores, this association did not remain statistically significant after correction for multiple comparisons using the Benjamini–Hochberg false discovery rate procedure. A possible hypothesis is that macrovascular complications may have a greater impact on perceived energy levels than microvascular complications, consistent with previous reports of poorer patient-reported outcomes in patients with macrovascular disease [17]. Another possible hypothesis is that microvascular complications may be associated with a greater perceived impact of diabetes on daily life because complications such as neuropathy, retinopathy, and nephropathy often require continuous self-management and lifestyle adaptation despite relatively preserved mobility. This interpretation is consistent with previous reports indicating that microvascular and macrovascular complications may impair quality of life through different pathways, with microvascular complications contributing more to disease-related burden and macrovascular complications predominantly affecting physical functioning [13,17]. However, the present findings did not remain statistically significant after correction for multiple comparisons and should therefore be regarded as exploratory. The persistence of the difference in DSMQ2 after adjustment suggests that specific diabetes self-management behaviors may differ according to complication profile. This finding is plausible because the DSMQ-derived item assessing adherence to the recommended meal plan was developed to assess diabetes self-care behaviors associated with glycemic control, with individual items reflecting distinct domains of self-management rather than only overall treatment adherence [11].

Patients with microvascular complications may adopt healthier dietary behaviors, including greater fruit consumption, as part of ongoing lifestyle adaptations following the diagnosis of chronic complications. Likewise, emotional eating may represent an important aspect of diabetes self-management in this population. However, neither the Fruit consumption nor the Stress-related eating scores remained statistically significant after correction for multiple comparisons, and these observations should therefore be considered exploratory. Previous research has shown that psychological distress and diabetes-related emotional burden are closely linked to eating behaviors and self-management practices, particularly among patients living with chronic diabetes complications [18]. Adjustment for covariate factors had only a modest influence on the overall findings. Blood glucose measurement and perceived disease control lost statistical significance after controlling for covariates, indicating that these associations were likely explained by confounding factors, whereas the remaining significant variables remained significantly associated with complication type after adjustment for demographic and clinical covariates. Among the exploratory dimensions identified, Functional Limitations emerged as the one most strongly associated with complication type. Patients with macrovascular complications showed greater functional impairment than those with microvascular complications, and this association remained significant after adjustment for demographic and clinical covariates. These findings are consistent with previous studies demonstrating that cardiovascular complications in diabetes are frequently associated with reduced exercise tolerance, impaired mobility, peripheral vascular disease, heart failure, fatigue, and reduced capacity to perform daily activities [10,19]. Therefore, greater functional limitations observed among patients with macrovascular complications may reflect a higher overall disease burden.

The lack of significant independent association between Emotional Burden Score and complication type may suggest that psychological distress is not limited to a specific complication profile, but rather reflects the overall chronic burden of living with diabetes. Previous studies have demonstrated that diabetes-related distress is associated with poorer self-care, reduced treatment adherence, and increased cardiovascular risk indicators [10]. However, distress appears to affect patients across different complication profiles, supporting the interpretation that emotional burden may represent an omnipresent dimension of chronic diabetes management rather than a distinguishing feature of either microvascular or macrovascular [10,20]. Glycemic Control Challenges Score was not independently associated with complication type, indicating that difficulties related to glycemic management may be shared across patients with different complication patterns [6,21]. Previous studies using the Diabetes Self-Management Questionnaire have also shown that self-management difficulties are multidimensional and not necessarily specific to a single complication category [11].

These results suggest that functional assessment should be integrated into routine diabetes care, particularly in patients with macrovascular complications. Previous studies have similarly emphasized the importance of combining metabolic management with physical-function assessment and patient-centered interventions to improve long-term outcomes and quality of life in diabetes populations [22,23,24]. Simple evaluation of mobility, daily activity limitations, fatigue, and work impairment may help identify patients with higher disease burden who may benefit from multidisciplinary interventions, including cardiometabolic optimization, rehabilitation, nutritional counseling, psychological support, and individualized physical activity programs. Previous evidence has highlighted that diabetic complications contribute substantially to functional impairment, reduced quality of life, and increased disability, underscoring the importance of their early detection and management [16,25].

This study has several limitations. First, its cross-sectional design does not allow causal inferences regarding the relationship between self-management dimensions and complication profiles. Second, some variables were based on self-reported data, which may introduce recall bias and subjective interpretation. Third, the study population was recruited from a single geographical region, which may limit generalizability. Fourth, although the models were adjusted for relevant demographic and clinical covariates, residual confounding cannot be excluded. Another limitation is the absence of objective clinical and biochemical indicators, such as HbA1c, fasting glucose, BMI, blood pressure, lipid profile, renal function, and albuminuria. Consequently, the Glycemic Control Challenges composite score reflects patients’ perceived difficulties with glycemic control rather than objectively measured metabolic control. Likewise, the emotional burden construct was derived from self-reported questionnaire items and should not be interpreted as a validated assessment of depression or diabetes-related distress. Future studies integrating patient-reported outcomes with objective clinical parameters would provide a more comprehensive evaluation of diabetes management. Therefore, the reported regression estimates should be interpreted as partially adjusted. In addition, patients with coexisting microvascular and macrovascular complications were excluded to enable comparison between two mutually exclusive clinical profiles. As a result, the study population does not fully represent the clinical heterogeneity of long-standing type 2 diabetes, and the findings may not be generalizable to patients with mixed complication profiles. Finally, the exploratory nature of the factor analysis requires validation in independent and larger cohorts. The questionnaire was translated into Romanian by the research team for the purposes of this study; however, no formal forward–backward translation, cultural adaptation, or psychometric validation was performed. Therefore, the study-derived questionnaire and composite scores should be interpreted with caution. The factor structure identified in the present study should be interpreted as exploratory and would benefit from confirmation using alternative correlation matrices in future validation studies. Some extracted factors were represented by a limited number of items and showed only moderate internal consistency. Therefore, these dimensions should be regarded as exploratory and require confirmation in independent samples using additional reliability indices. Internal consistency was evaluated using Cronbach’s alpha. Future validation studies should include additional reliability indices, including McDonald’s omega, to further evaluate the psychometric properties of the derived composite scores.

Future longitudinal and multicenter studies are necessary to confirm these findings and to clarify the temporal relationships between psychosocial, behavioral, and functional dimensions of diabetes self-management.

## 5. Conclusions

In conclusion, this study showed that diabetes self-management and disease-related experiences are multidimensional constructs encompassing emotional burden, dietary behavior, functional limitations, glycemic control challenges, glucose monitoring practices, and perceived general health. Three multidimensional composite domains were identified and used to characterize differences in patient-reported outcomes between complication groups: emotional burden, functional limitations, and glycemic control challenges. Patients with macrovascular complications reported greater functional impairment. Functional limitations emerged as the domain most strongly associated with complication type, being consistently more pronounced in patients with macrovascular complications. These findings highlight the importance of integrating functional, psychosocial, and behavioral assessment into comprehensive diabetes care. A multidimensional assessment of patients with different diabetes complication profiles may help clinicians identify distinct patterns of disease burden and patient-reported outcomes, although longitudinal studies are needed to determine the temporal relationships between these factors.

## Figures and Tables

**Table 1:** Characteristics of the patients with diabetic complications.

Characteristic	Microvascular Complications (n = 144)	Macrovascular Complications (n = 156)	*p*-Value
***Sex***			0.148
Female	72 (50.0%)	91 (58.3%)	
Male	72 (50.0%)	65 (41.7%)	
***Residence area***			0.477
Urban	64 (44.4%)	63 (40.4%)	
Rural	80 (55.6%)	93 (59.6%)	
***Marital status***			
Unmarried	6 (4.2%)	9 (5.8%)	0.816
Married	96 (66.7%)	107 (68.6%)	
Divorced	10 (6.9%)	8 (5.1%)	
Widower	32 (22.2%)	32 (20.5%)	
***Education level***			0.538
Primary school	8 (5.6%)	5 (3.2%)	
Secondary school	57 (39.6%)	58 (37.2%)	
High school	60 (41.7%)	76 (48.7%)	
Post high school	13 (9.0%)	9 (5.8%)	
University	6 (4.2%)	8 (5.1%)	
***Treatment type***			0.012
Diet only	2 (1.4%)	1 (0.6%)	
Oral antidiabetic medication	80 (55.6%)	115 (73.7%)	
Insulin	18 (12.5%)	11 (7.1%)	
Mixed treatment	44 (30.6%)	29 (18.6%)	
Age *	65.50 (58.50–71.00)	68.00 (61.00–73.00)	0.095
Diabetes duration *	10.00 (4.50–18.00)	9.00 (4.00–15.00)	0.156

* Expressed as median and interquartile range.

**Table 2:** Differences between groups with different diabetes complications.

Variable	Mean (Unadjusted)		Mean (Adjusted)		Unadjusted			Adjusted			
	Microvascular	Macrovascular	Microvascular	Macrovascular	*F*	*p*	*η^2^p*	*F*	*p*	FDR-Adjusted *p*	*η^2^p*
DSMQ1 ^a^	2.225	2.410	2.266	2.362	1.429	0.233	0.005	0.378	0.539	0.702	0.001
DSMQ2 ^a^	3.077	2.724	3.096	2.714	8.382	0.004	0.028	9.097	0.003	0.043	0.031
DSMQ3 ^a^	2.310	2.513	2.308	2.516	2.703	0.101	0.009	2.598	0.108	0.411	0.009
DSMQ4 ^a^	2.859	2.712	2.866	2.705	0.988	0.321	0.003	1.100	0.295	0.568	0.004
DSMQ5 ^a^	1.401	1.269	1.407	1.267	1.841	0.176	0.006	1.935	0.165	0.411	0.007
DSMQ6 ^a^	3.085	3.122	3.062	3.156	0.064	0.801	0.000	0.385	0.536	0.702	0.001
DSMQ7 ^a^	1.183	1.179	1.183	1.181	0.003	0.956	0.000	0.002	0.967	0.967	0.000
DSMQ8 ^a^	2.155	1.962	2.155	1.969	2.198	0.139	0.007	1.947	0.164	0.411	0.007
Blood glucose measurement ^a^	1.986	1.788	1.959	1.819	4.820	0.029	0.016	2.439	0.119	0.411	0.008
Hypoglycemia ^b^	1.704	1.827	1.705	1.825	5.530	0.019	0.018	4.895	0.028	0.150	0.017
Hyperglycemia ^b^	1.197	1.212	1.198	1.212	0.020	0.889	0.000	0.016	0.899	0.967	0.000
Monitoring importance ^c^	8.923	8.917	8.926	8.919	0.001	0.971	0.000	0.002	0.967	0.967	0.000
Confidence self-management ^c^	7.289	7.635	7.318	7.603	3.335	0.069	0.011	2.219	0.137	0.411	0.008
Daily meals ^a^	2.782	2.827	2.768	2.838	0.497	0.481	0.002	1.127	0.289	0.568	0.004
Snacks ^a^	3.338	3.506	3.344	3.497	3.611	0.058	0.012	2.844	0.093	0.411	0.010
Regular meal schedule ^d^	2.359	2.455	2.371	2.440	1.197	0.275	0.004	0.612	0.435	0.652	0.002
Vegetable consumption ^d^	1.472	1.301	1.455	1.320	3.400	0.066	0.011	2.006	0.158	0.411	0.007
Fruit consumption ^d^	1.570	1.359	1.564	1.369	8.430	0.004	0.028	6.785	0.010	0.072	0.023
Sweets consumption ^a^	2.423	2.506	2.423	2.508	1.109	0.293	0.004	1.062	0.304	0.568	0.004
Sugar-sweetened beverages ^d^	2.246	2.256	2.259	2.247	0.021	0.884	0.000	0.030	0.863	0.967	0.000
Carbohydrate awareness ^e^	2.577	2.647	2.571	2.651	0.371	0.543	0.001	0.457	0.500	0.702	0.002
Diet adjustment based on glucose ^e^	2.338	2.340	2.295	2.375	0.000	0.988	0.000	0.427	0.514	0.702	0.001
Knowledge of high-glycemic foods ^d^	1.408	1.340	1.387	1.361	0.621	0.431	0.002	0.085	0.770	0.919	0.000
Stress-related eating ^e^	2.880	2.551	2.887	2.532	3.589	0.059	0.012	4.064	0.045	0.215	0.014
Difficulty following diet ^e^	3.021	3.077	3.021	3.071	0.177	0.674	0.001	0.141	0.708	0.887	0.000
Perceived diet adequacy ^e^	2.451	2.487	2.454	2.487	0.081	0.776	0.000	0.068	0.794	0.919	0.000
Nutrition education ^b^	1.007	1.064	0.996	1.074	0.714	0.399	0.002	1.235	0.267	0.568	0.004
Source of education ^a^	1.007	1.000	1.007	1.000	1.099	0.295	0.004	0.971	0.325	0.586	0.003
Perceived usefulness of education ^c^	9.408	9.564	9.412	9.558	1.365	0.244	0.005	1.114	0.292	0.568	0.004
General health status ^e^	3.831	3.878	3.818	3.888	0.226	0.635	0.001	0.483	0.488	0.702	0.002
Severe limitations ^d^	1.789	2.083	1.785	2.092	9.764	0.002	0.032	10.336	0.001	0.022	0.035
Moderate limitations ^d^	1.845	2.109	1.851	2.109	8.432	0.004	0.028	7.850	0.005	0.050	0.027
Mobility limitations ^d^	1.873	2.282	1.894	2.269	18.451	<0.001	0.059	14.817	<0.001	0.022	0.049
Work impairment ^b^	1.486	1.519	1.489	1.520	0.283	0.595	0.001	0.238	0.626	0.790	0.001
Fatigue ^e^	4.113	3.987	4.111	3.983	0.608	0.436	0.002	0.598	0.440	0.652	0.002
Sadness ^e^	4.106	4.051	4.115	4.037	0.140	0.708	0.000	0.275	0.600	0.780	0.001
Energy level ^e^	2.542	2.250	2.558	2.240	6.560	0.011	0.022	7.227	0.008	0.069	0.024
Social life impairment ^e^	3.423	3.333	3.424	3.322	0.436	0.509	0.001	0.538	0.464	0.702	0.002
Perceived disease severity ^b^	1.021	1.006	1.020	1.008	0.804	0.371	0.003	0.534	0.465	0.702	0.002
Perceived disease control ^b^	1.261	1.141	1.243	1.158	4.018	0.046	0.013	2.025	0.156	0.411	0.007
Diabetes impact on life ^e^	3.507	3.244	3.508	3.233	5.155	0.024	0.017	5.280	0.022	0.135	0.018
Diabetes-related stress level ^c^	6.951	6.462	6.922	6.468	2.284	0.132	0.008	1.875	0.172	0.411	0.006
Support and information need ^f^	1.514	1.532	1.491	1.557	0.012	0.913	0.000	0.152	0.697	0.887	0.001

DSMQ1—regular monitoring of blood glucose according to medical recommendations; DSMQ2—adherence to the recommended meal plan; DSMQ3—frequent consumption of sweets or high-carbohydrate foods (reverse scored); DSMQ4—engagement in regular physical activity; DSMQ5—adherence to prescribed medication treatment; DSMQ6—forgetting to take medication or administer insulin (reverse scored); DSMQ7—attendance at recommended periodic medical check-ups; DSMQ8—perceived effectiveness of diabetes self-management. Data are presented as unadjusted and adjusted estimated marginal means. Adjusted means were obtained using analysis of covariance (ANCOVA), controlling for age, sex, residence area, marital status, educational level, diabetes duration, and diabetes treatment. *F* and *p* values correspond to the overall effect of complication group before (unadjusted) and after adjustment for the covariates. For the adjusted analyses, *p*-values were corrected for multiple testing using the Benjamini–Hochberg false discovery rate (FDR) procedure; values ranged between: ^a^ 1 and 4, ^b^ 1 and 2, ^c^ 1 and 10, ^d^ 1 and 3, ^e^ 1 and 5, ^f^ 1 and 6.

**Table 3:** Correlations between scores.

Item	Emotional Burden Score	Functional Limitations Score	Glycemic Control Challenges Score
Emotional Burden Score	1.000	−0.210 **	0.205 **
Functional Limitations Score	−0.210 **	1.000	−0.122 **
Glycemic Control Challenges Score	0.205 **	−0.122 **	1.000

** Correlation is significant at the 0.01 level (2-tailed).

**Table 4:** Logistic regression analysis results for the scores developed.

Variable	Model	B (SE)	OR (95% CI)	*p*-Value
Emotional Burden Score	Unadjusted	0.203 (0.113)	1.23 (0.98–1.53)	0.073
	Adjusted *	0.218 (0.121)	1.24 (0.98–1.58)	0.071
Functional Limitations Score	Unadjusted	−0.668 (0.192)	0.51 (0.35–0.75)	<0.001
	Adjusted *	−0.687 (0.205)	0.50 (0.34–0.75)	0.001
Glycemic Control Challenges Score	Unadjusted	−0.189 (0.227)	0.828 (0.53–1.292)	0.406
	Adjusted *	0.230 (0.243)	1.80 (0.49–1.28)	0.344

* Adjusted model covariates: age, sex, residence area, marital status, education, diabetes duration, and treatment type.

## Data Availability

The data are available upon request from the corresponding author.

## Supplementary Materials

The following supporting information can be downloaded at: https://www.mdpi.com/article/10.3390/jcm15156125/s1, Table S1: Pattern Matrix of exploratory factor analysis; Table S2: FA results, Table S3: Evaluation of the regression models; Table S4: Questionnaire legend and score construction.
