# Supplementary material for: Exploring Multidimensional Diabetes Self-Management and Health-Related Functioning in Relation to Vascular Complications
Source: J Clin Med. 2026 Aug 6;15(15):6125. doi: 10.3390/jcm15156125 (PMC13467379; doi:10.3390/jcm15156125)
Supplement: Supplementary file 1 [file jcm-15-06125-s001.zip › SUPPLEMENTARY MATERIAL.pdf]

SUPPLEMENTARY MATERIAL

Table S1. Pattern Matrix of exploratory factor analysis [a]

Item	Factor					
	1	2	3	4	5	6
DSMQ1	-.060	-.129	-.045	.101	-.888	.057
DSMQ2	-.049	-.659	-.066	.067	-.042	-.121
DSMQ3	-.027	.529	.124	.114	.015	.067
DSMQ4	.352	-.363	-.191	-.268	.039	.127
DSMQ5	.123	-.005	.118	.567	-.124	-.003
DSMQ6	-.240	-.130	.059	-.456	.080	.273
DSMQ7	.033	-.013	-.029	.407	-.114	-.016
DSMQ8	-.080	-.371	-.027	-.057	-.054	.239
blood glucose measurement	.135	.053	.076	-.050	.847	-.124
hypoglycemia	-.029	-.063	.057	-.325	-.088	-.013
hyperglycemia	.093	.082	.045	.544	-.021	.046
monitoring importance	.077	.213	.072	-.240	.240	.112
confidence self management	-.076	.166	.078	-.448	-.026	-.195
daily meals	.196	.050	.014	.194	-.023	.032
snacks	.582	.038	.161	-.186	-.145	.041
regular meal schedule	.542	-.106	.047	-.316	-.144	-.011
vegetable consumption	-.224	-.270	-.049	-.066	.112	.064
fruit consumption	-.179	-.378	-.012	.295	.094	.189
sweets consumption	-.162	.492	-.023	-.071	-.008	.316
sugar sweetened beverages	-.167	.329	.015	.218	-.056	.092
carbohydrate awareness	.091	.669	-.071	.061	.021	-.017
diet adjustment based on glucose	-.050	.637	-.180	-.019	.158	-.047
knowledge of high glycemic foods	-.193	-.169	-.001	.214	.133	.249
stress related eating	.595	.037	-.046	.163	.109	-.226
difficulty following diet	.345	-.252	-.021	.017	.002	-.392
perceived diet adequacy	-.217	.390	-.043	-.128	.136	-.239
nutrition education	-.080	-.071	-.075	-.056	.056	-.257
source of education	-.055	-.010	-.099	-.014	.003	-.011
perceived usefulness of education	.309	.331	.061	.045	-.107	.023
general health status	.343	-.015	-.134	-.068	.039	.440
severe limitations	-.070	-.008	.705	-.025	.015	.055
moderate limitations	-.013	-.129	1.018	.046	.083	.108
mobility limitations	.044	.010	.897	-.042	.003	.182
work impairment	-.066	.072	.488	.027	.067	-.164
fatigue	.772	.071	-.060	.110	-.017	.316
sadness	.814	.090	-.063	.083	.017	.300
energy level	-.070	.109	-.122	.008	.104	-.273
social life impairment	.707	.085	.095	.148	.092	.122
perceived disease severity	-.130	.001	.057	-.054	.089	-.062
perceived disease control	-.279	-.087	-.263	-.042	-.012	.154
diabetes impact on life	.518	.053	-.110	.064	.098	.009
diabetes related stress level	.474	-.244	-.003	.093	.298	-.132
support and information need	-.435	.213	-.077	-.036	-.014	.024

Extraction Method: Principal Axis Factoring.
Rotation Method: Promax with Kaiser Normalization.
a. Rotation converged in 8 iterations.

Medical Nutrition Therapy and Quality of Life in Patients with Type 2 Diabetes Mellitus - Questionnaire

Instructions for Participants

This questionnaire is anonymous and is part of a scientific study conducted for academic purposes. Please answer all questions honestly by selecting the option that best describes your situation. There are no right or wrong answers.

Estimated completion time: 20–25 minutes.

SOCIO-DEMOGRAPHIC DATA

Age: years

Sex: ☐ Female ☐ Male

Place of residence: ☐ Urban ☐ Rural

Marital status: ☐ Single ☐ Married ☐ Divorced ☐ Widowed

Education level: ☐ Primary ☐ Secondary ☐ High school ☐ Post-secondary ☐ University ☐ Postgraduate

Occupation:

Do you have someone who supports you in managing diabetes? ☐ Yes ☐ No

If yes, who?

CLINICAL DATA REGARDING DIABETES

Type of diabetes: ☐ Type 1 ☐ Type 2 ☐ Gestational

Year of diagnosis:

Duration of disease: years

Current treatment (multiple answers possible): ☐ Diet ☐ Oral antidiabetics ☐ Insulin ☐ Combined

Diabetes-related complications (if any): ☐ Retinopathy ☐ Neuropathy ☐ Nephropathy ☐ Cardiovascular disease ☐ Others:

DIABETES SELF-MANAGEMENT (DSMQ adaptation)

Please indicate to what extent the following statements apply to you over the last 8 weeks:

Statement	4<br>Applies to me very much	3<br>Applies to me to a considerable extent	2<br>Applies to me to some extent	1<br>Does not apply to me
1. I regularly monitor my blood glucose according to medical recommendations.	☐	☐	☐	☐
2. I follow the recommended meal plan.	☐	☐	☐	☐
3. I frequently consume sweets or foods high in carbohydrates. (reverse item)	☐	☐	☐	☐
4. I engage in regular physical activity.	☐	☐	☐	☐
5. I follow my medication treatment exactly as prescribed.	☐	☐	☐	☐
6. I forget to take my medication or administer insulin. (reverse item)	☐	☐	☐	☐

7. I attend recommended periodic medical check-ups.	☐	☐	☐	☐
8. I consider my diabetes self-management to be effective.	☐	☐	☐	☐

SELF-CARE BEHAVIORS

1. How many times a day do you measure your blood glucose? ☐ I do not measure ☐ Once ☐ 2–3 times ☐ >3 times

2. Have you had episodes of hypoglycemia in the last 3 months? ☐ Yes ☐ No

3. Have you had frequent episodes of hyperglycemia? ☐ Yes ☐ No

4. How important do you consider daily blood glucose monitoring? (0–10):

5. How confident are you in your ability to manage diabetes? (0–10):

NUTRITION AND EATING BEHAVIORS

A. Meal Organization

1. How many main meals do you have per day? ☐ 1 ☐ 2 ☐ 3 ☐ >3

2. Do you consume snacks between meals? ☐ Never ☐ Rarely ☐ Sometimes ☐ Frequently

3. Do you follow relatively fixed meal times? ☐ Yes ☐ Partially ☐ No

B. Diet Quality

1. Do you consume fresh or cooked vegetables daily? ☐ Yes ☐ Sometimes ☐ No

2. Do you consume fruit daily? ☐ Yes ☐ 1–3 times/week ☐ Rarely

3. How often do you consume sweets or sugary products? ☐ Daily ☐ Several times/week ☐ Rarely ☐ Never

4. Do you consume sweetened beverages (soft drinks, energy drinks)? ☐ Daily ☐ Occasionally ☐ Never

C. Portion and Carbohydrate Control

1. I pay attention to the amount of carbohydrates I consume. ☐ Not at all ☐ A little ☐ Moderately ☐ Much ☐ Very much

2. I adjust my diet according to blood glucose values. ☐ Never ☐ Rarely ☐ Sometimes ☐ Often ☐ Always

3. I know which foods quickly increase my blood glucose. ☐ Yes ☐ Partially ☐ No

D. Eating Behavior and Emotions

1. I eat more when I am stressed or tired. ☐ Never ☐ Rarely ☐ Sometimes ☐ Often ☐ Always

2. I have difficulty following the recommended diet. ☐ Never ☐ Rarely ☐ Sometimes ☐ Often ☐ Always

3. I consider my diet adequate for diabetes control. ☐ Not at all ☐ A little ☐ Moderately ☐ Much ☐ Very much

E. Nutritional Support and Education

1. Have you received specialized nutritional education? ☐ Yes ☐ No

2. If yes, from whom? ☐ Doctor ☐ Nutritionist ☐ Nurse ☐ Other

3. How useful do you consider the nutritional education received? (0–10):

QUALITY OF LIFE – HEALTH STATUS (SF-36 adaptation)

In general, how would you rate your health status? ☐ Excellent ☐ Very good ☐ Good ☐ Fair ☐ Poor
To what extent does your health condition limit your daily activities?

a) Intense physical activities ☐ A lot ☐ A little ☐ Not at all

b) Moderate activities ☐ A lot ☐ A little ☐ Not at all

c) Walking ☐ A lot ☐ A little ☐ Not at all

During the last 4 weeks, has your health condition affected your work or daily activities? ☐ Yes ☐ No

Severe fatigue ☐ Never ☐ Rarely ☐ Sometimes ☐ Often ☐ All the time

Sadness ☐ Never ☐ Rarely ☐ Sometimes ☐ Often ☐ All the time

Energy and vitality ☐ Never ☐ Rarely ☐ Sometimes ☐ Often ☐ All the time

To what extent does diabetes affect your social life? ☐ Not at all ☐ A little ☐ Moderately ☐ Much ☐ Very much

PSYCHO-EMOTIONAL ASPECTS AND ADAPTATION

Do you consider diabetes a serious disease? ☐ Yes ☐ No

Do you believe you have control over the disease? ☐ Yes ☐ No

Diabetes affects my quality of life. ☐ Not at all ☐ A little ☐ Moderately ☐ Much ☐ Very much
Stress level associated with diabetes management (0–10):

GOALS AND NEEDS

In which areas would you like more support/information? (check all that apply)
☐ Nutrition ☐ Physical activity ☐ Medication ☐ Blood glucose monitoring ☐ Stress management ☐ Prevention of complications

What do you consider to be the greatest difficulty in managing diabetes?

Thank you for your participation!
